# Interim Recommendations for Use of Bivalent mRNA COVID-19 Vaccines for Persons Aged ≥6 Months — United States, April 2023

**DOI:** 10.15585/mmwr.mm7224a3

**Published:** 2023-06-16

**Authors:** Danielle L. Moulia, Megan Wallace, Lauren E. Roper, Monica Godfrey, Hannah G. Rosenblum, Ruth Link-Gelles, Amadea Britton, Matthew F. Daley, Sarah Meyer, Katherine E. Fleming-Dutra, Sara E. Oliver, Evelyn Twentyman

**Affiliations:** ^1^National Center for Immunization and Respiratory Diseases, CDC; ^2^Institute for Health Research, Kaiser Permanente Colorado, Denver, Colorado.

Throughout the national public health emergency declared in response to the COVID-19 pandemic, CDC, guided by the Advisory Committee on Immunization Practices (ACIP), has offered evidence-based recommendations for the use of COVID-19 vaccines in U.S. populations after each regulatory action by the Food and Drug Administration (FDA). During August 2022–April 2023, FDA amended its Emergency Use Authorizations (EUAs) to authorize the use of a single, age-appropriate, bivalent COVID-19 vaccine dose (i.e., containing components from the ancestral and Omicron BA.4/BA.5 strains in equal amounts) for all persons aged ≥6 years, use of bivalent COVID-19 vaccine doses for children aged 6 months–5 years, and additional bivalent doses for immunocompromised persons and adults aged ≥65 years (*1*). ACIP voted in September 2022 on the use of the bivalent vaccine, and CDC made recommendations after the September vote and subsequently, through April 2023, with input from ACIP. This transition to a single bivalent COVID-19 vaccine dose for most persons, with additional doses for persons at increased risk for severe disease, facilitates implementation of simpler, more flexible recommendations. Three COVID-19 vaccines are currently available for use in the United States and recommended by ACIP: 1) the bivalent mRNA Pfizer-BioNTech COVID-19 vaccine, 2) the bivalent mRNA Moderna COVID-19 vaccine, and 3) the monovalent adjuvanted, protein subunit-based Novavax COVID-19 vaccine.* As of August 31, 2022, monovalent mRNA vaccines based on the ancestral SARS-CoV-2 strain are no longer authorized for use in the United States (*1*).

Since June 2020, ACIP has convened 35 public meetings to review data relevant to the potential use of COVID-19 vaccines.^†^ The ACIP COVID-19 Vaccine Work Group, comprising experts in adult and pediatric medicine, infectious diseases, vaccinology, vaccine safety, public health, and ethics, has met weekly to review COVID-19 surveillance data, evidence for immunogenicity, efficacy, postauthorization effectiveness, safety of COVID-19 vaccines, and implementation considerations. To assess the evidence for benefits and harms associated with use of bivalent vaccines, and to guide deliberations, ACIP used the Evidence to Recommendations (EtR) Framework.^§^ Within this framework, ACIP considered the importance of COVID-19 as a public health problem, including during the Omicron-predominant era, as well as issues of resource use, benefits and harms, patients’ values and preferences, acceptability, feasibility, and equity related to use of the vaccines. ACIP held three public meetings on September 1, 2022, February 24, 2023, and April 19, 2023, to discuss bivalent vaccine policy using the EtR Framework. ACIP voted on adult bivalent doses on September 1, 2022. Authorization for bivalent vaccines was subsequently extended to additional age groups, and CDC updated recommendations, guided by February 24, 2023, and April 19, 2023, input from ACIP (Box). To better protect against the Omicron variant, which emerged in November 2021, ACIP recommended a dose of bivalent mRNA vaccine (containing mRNA encoding the spike protein from both the ancestral SARS-CoV-2 and Omicron BA.4/BA.5 SARS-CoV-2 variants) in September 2022 (*2*). Among persons who had only received monovalent COVID-19 vaccines, bivalent COVID-19 vaccines have provided additional protection against infection and COVID-19–associated hospitalization; however, that protection might wane over time (*3*). From September 2022 to March 2023, vaccine effectiveness (VE) against emergency department and urgent care visits by adults aged 18–64 years waned from 53% (95% CI = 48%–58%) at 7–59 days after receipt of a bivalent dose to 42% (95% CI = 35%–47%) at 60–119 days. Protection against hospitalization among adults aged 18–64 years without an immunocompromising condition waned from 68% (95% CI = 53%–79%) at 7–59 days to 27% (95% CI = 2%–46%) at 60–119 days (*3*).

BOX **Timeline of COVID-19 bivalent vaccine authorizations, by Food and Drug Administration and CDC vaccine recommendations — United States, August 2022–April 2023**

**August and September 2022**

FDA authorizes and CDC recommends, with a single ACIP vote, 1) a single dose of Pfizer-BioNTech bivalent vaccine for persons aged ≥12 years 2 or more months after receipt of a primary series or previous monovalent booster dose and 2) a single dose of Moderna bivalent vaccine for adults aged ≥18 years 2 or more months after receipt of a primary series or previous monovalent booster dose.

**October 2022**

FDA authorizes and CDC recommends, with ACIP input, a single dose of Pfizer-BioNTech bivalent vaccine for children aged 5–11 years 2 or more months after receipt of a primary series or previous monovalent booster dose.

**December 2022**

FDA authorizes and CDC recommends, with ACIP input, a single dose of Moderna bivalent vaccine for children aged 6 months–5 years 2 or more months after receipt of a primary series.

FDA authorizes and CDC recommends, with ACIP input, a single dose of Pfizer-BioNTech bivalent vaccine for children aged 6 months–4 years as the third dose in a primary series 8 or more weeks after receipt of 2 monovalent doses of Pfizer-BioNTech vaccine.

**March 2023**

FDA authorizes and CDC recommends, with ACIP input, a single dose of Pfizer-BioNTech bivalent vaccine for children aged 6 months–4 years who received 3 monovalent doses of Pfizer-BioNTech as a primary series.

**April 2023**

FDA authorizes and CDC recommends, with ACIP input, a single dose of bivalent vaccine for all persons aged ≥6 years who are unvaccinated or 2 or more months after receipt of a previous monovalent dose.

FDA authorizes and CDC recommends, with ACIP input, at the time of initial vaccination (depending on vaccine product) 2 or 3 doses of bivalent vaccine for children aged 6 months–4 years and 1 or 2 doses of bivalent vaccine for children aged 5 years.

FDA authorizes and CDC recommends, with ACIP input, that persons aged ≥65 years may receive a single additional bivalent vaccine dose 4 or more months after receipt of their first bivalent dose.

FDA authorizes and CDC recommends, with ACIP input, that persons aged ≥6 months who are moderately or severely immunocompromised may receive an optional additional bivalent dose 2 or more months after the most recent bivalent dose and additional bivalent doses as needed.

**Abbreviations:** ACIP = Advisory Committee on Immunization Practices; FDA = Food and Drug Administration.

As of May 6, 2023, COVID-19–associated hospitalization rates were highest among adults aged ≥65 years (9.5 per 100,000 persons).^¶^ Bivalent booster doses are shown to provide the highest protection against hospitalization among adults, with protection sustained through at least 179 days against critical outcomes, including intensive care unit admission or in-hospital death (*4*). However, only 17% of the U.S. population overall and 43.3% of adults aged ≥65 years have received a bivalent dose.** Primary series coverage (i.e., receipt of a complete COVID-19 vaccination series) follows a similar pattern: it is highest among older adults and lowest among young children.

## Recommendations for Use of Bivalent COVID-19 Vaccines in Persons Aged ≥6 Years Without Immunocompromising Conditions

On April 18, 2023, FDA authorized, and on April 20, 2023, CDC recommended a single, age-appropriate bivalent mRNA dose for unvaccinated persons aged ≥6 years without moderate or severe immunocompromise. Previously vaccinated persons without moderate or severe immunocompromise were recommended to receive the vaccine ≥2 months after receipt of any monovalent vaccine dose (Table).

**TABLE:** COVID-19 vaccines recommended for persons aged ≥6 months, by immunocompromise status and age group — CDC, United States, April 2023*

Immunocompromise status	Age group	Recommendation
Not moderately or severely immunocompromised	6 mos–5 yrs	At the time of initial vaccination, depending on vaccine product: 2 or 3 doses of bivalent vaccine for children aged 6 mos–4 yrs (Pfizer-BioNTech); and 1 or 2 doses of bivalent vaccine for children aged 5 yrs (Moderna)^†^
	≥6 yrs	A single bivalent dose
	≥65 yrs	A single bivalent dose and 1 additional, optional, bivalent dose
Moderately or severely immunocompromised	≥6 mos	A single bivalent dose and additional, optional, bivalent doses as needed

* https://www.cdc.gov/vaccines/covid-19/clinical-considerations/interim-considerations-us.html

† https://www.cdc.gov/vaccines/covid-19/clinical-considerations/interim-considerations-us.html#not-immunocompromised

CDC’s recommendation was based on input from ACIP during public meetings held on February 24, 2023, and April 19, 2023. At these meetings, discussions were guided by clinical trial data demonstrating that bivalent vaccines induce an immune response when administered as a primary series. Immunogenicity data demonstrated that a primary series of an Omicron BA.1-containing bivalent vaccine induced neutralization titers against BA.1 that were approximately 25 times those induced by the original monovalent vaccine^††^ (*5*). The percentage of patients reporting solicited systemic and local reactogenicity after receiving the BA.1-containing vaccine was similar to or less than the percentage reporting these reactions after a receipt of a monovalent vaccine.^§§^ Unsolicited adverse events generally represented illnesses and commonly reported events during infancy and childhood (*5*).

CDC recommendations were also guided by ACIP discussions of seroprevalence data indicating that, for most older children, adolescents, and adults, future doses will provide an additional boost after previous infection, previous vaccination, or both. In a March–December 2022 nationwide seroprevalence study conducted among children aged 6 months–17 years, most children and adolescents had evidence of infection-induced immunity; prevalence of infection-induced immunity was highest among persons aged 5–11 and 12–17 years (93%) and lowest among children aged 6–11 months (63%) (*6*). Most adults also had preexisting antibodies against SARS-CoV-2. In a January–March 2022 seroprevalence study of adult blood donors aged ≥16 years, 96% had evidence of immunity from either previous infection, previous vaccination, or both (*7*).

A rare risk for myocarditis and pericarditis has been identified after receipt of monovalent mRNA COVID-19 vaccines, primarily in adolescent and young adult males. Because data are limited, the risk for myocarditis or pericarditis after receipt of a bivalent dose is not known; however, preliminary estimates suggest that the risk is lower than that observed after a second primary series monovalent dose (*8*). Higher rates of myocarditis have also been associated with a shorter interval between doses (*9*). Because of the small number of doses administered among adolescent and young adult males, estimating the incidence of myocarditis after a bivalent dose was not possible; however, only a single case of myopericarditis has been observed in the Vaccine Safety Datalink (a postauthorization vaccine safety monitoring system) during the 7 days after receipt of a bivalent dose in a male aged 18–29 years (*8*).

Recommendations from CDC were also guided by ACIP discussions of the benefits (i.e., reduction in the number of hospitalizations, intensive care unit admissions, and the number of deaths prevented) per 1 million primary series and bivalent vaccine doses administered, stratified by both age group and interval between primary series completion and receipt of a first bivalent dose. Benefits of a primary series and bivalent dose were seen among all age groups and at all intervals; however, the largest observed benefits were among the oldest age groups and those with the longest interval (i.e., ≥11 months) between completion of the primary series and receipt of the bivalent dose (*10*). Regular review of safety data, including myocarditis and pericarditis risk after bivalent doses, will continue in national safety surveillance systems.

## Recommendations for Use of Bivalent COVID-19 Vaccine for Children Aged 6 Months–5 Years

During December 2022–April 2023, FDA amended multiple authorizations for bivalent mRNA vaccines for children aged 6 months–5 years. During this period, CDC updated recommendations, with input from ACIP, for children in this age group for use of bivalent doses based on a child’s vaccination history (Table).

Among children aged 6 months–4 years, either mRNA vaccine may be used; however, all doses administered to a given child must be from the same manufacturer. Among those receiving Moderna vaccine, ≥2 doses are authorized, including ≥1 bivalent vaccine dose. Among those receiving Pfizer-BioNTech vaccine, ≥3 doses are authorized, including ≥1 bivalent vaccine dose.

Based on FDA authorizations, unvaccinated children aged 5 years are authorized to receive 2 doses of Moderna bivalent vaccine (with 4–8 weeks between doses) or 1 dose of Pfizer-BioNTech vaccine. Children aged 5 years who received 1 or 2 doses of monovalent Moderna vaccine are authorized to receive 1 dose of either the bivalent Moderna or Pfizer-BioNTech vaccine.^¶¶^ Those who received ≥1 doses of monovalent Pfizer-BioNTech vaccine are authorized to receive ≥1 bivalent Pfizer-BioNTech vaccine doses.

CDC recommendations for pediatric immunization were guided by ACIP discussion of studies of bivalent vaccine given as a primary series in children, as well as booster doses among adults, demonstrating that a bivalent dose of either Moderna or Pfizer-BioNTech vaccine broadens the immune response in persons who have received a primary series and a previous monovalent booster dose (*5*). Compared with a monovalent booster dose (based on the ancestral SARS-CoV-2 strain), the immune response to Omicron was superior and that to the ancestral strain was noninferior among bivalent vaccine booster dose recipients. Monovalent booster doses of Moderna COVID-19 vaccines were studied in a clinical trial of 145 children aged 17 months–5 years who had received a Moderna primary series 8–13 months previously. Antibody levels after receipt of the monovalent booster dose in a subset of 56 children without previous SARS-CoV-2 infection were four times higher than were levels after the primary series in 294 young adults (*11*). Reactogenicity was similar to that observed after receipt of booster doses in other age groups. In a subset of 60 Pfizer-BioNTech pediatric trial participants aged 6 months–4 years who received a single bivalent Pfizer-BioNTech vaccine dose after completion of a 3-dose monovalent primary series, Omicron BA.4/BA.5–specific antibodies were higher compared with those among children who completed the 3-dose primary series of monovalent Pfizer-BioNTech vaccine and did not receive the bivalent booster dose. The bivalent dose was generally well-tolerated, with a lower frequency of postvaccination local and systemic reactions than previously observed in this age group with monovalent doses; no new or concerning safety findings were identified (*12*).

## Additional Bivalent COVID-19 Doses for Adults Aged ≥65 Years and for Persons Aged ≥6 Months Who Are Moderately or Severely Immunocompromised

In April 2023, FDA granted an EUA for additional bivalent doses for adults aged ≥65 years and for persons aged ≥6 months with immunocompromise. Adults aged ≥65 years have the option to receive 1 additional bivalent vaccine dose ≥4 months after receipt of the most recent bivalent dose (Table). Persons aged ≥6 months who are moderately or severely immunocompromised have the option to receive ≥1 additional bivalent doses ≥2 months after receipt of the most recent bivalent dose and additional bivalent mRNA doses, as indicated, based on individual circumstances and clinical judgment.*** The option to receive ≥1 additional bivalent mRNA vaccine doses may be based on the clinical judgment of a health care provider, a person’s risk for severe COVID-19 because of the presence of underlying medial conditions and age, and personal preference and circumstances.

CDC made recommendations based on ACIP discussions of VE and clinical epidemiology of COVID-19 among moderately or severely immunocompromised persons and adults aged ≥65 years. Effectiveness of a bivalent vaccine booster dose against hospitalization in adults aged ≥18 years with immunocompromising conditions was 30% (95% CI = 12%–44%) at 7–59 days postvaccination and 31% (95% CI = 4%–50%) at 120–179 days (*3*). Among adults aged ≥65 years, waning of absolute VE has been noted after receipt of a bivalent dose. Effectiveness of bivalent vaccines against COVID-19–associated emergency department or urgent care encounters among immunocompetent adults aged ≥65 years declined from 61% (95% CI = 57%–64%) 7–59 days after vaccination to 25% (95% CI = 16%–34%) at 120–179 days (*3*). VE against COVID-19–associated hospitalization declined from 64% (95% CI = 59%–69%) 7–59 days after vaccination to 39% (95% CI = 26%–50%) at 120–179 days (*3*).

## Implementation Considerations

Before the authorization of a bivalent dose for most persons, 11 mRNA COVID-19 vaccine products were licensed or authorized for use. Authorization of a bivalent dose for most persons reduced the total number of vaccine products to five and eliminated vials that appear similar (i.e., look-alike vials) (*13*); these recommendations will thereby simplify implementation for COVID-19 vaccine providers. Reducing the number of products will expand providers’ storage space and, in conjunction with the elimination of look-alike vials, might reduce vaccine administration errors.

The transition from a monovalent primary series to a single bivalent dose for most persons, and additional bivalent doses for populations at higher risk for severe disease, allows the COVID-19 vaccination program to progress toward simpler, more flexible, evidence-based recommendations. COVID-19 vaccination remains critical to protecting against serious consequences of COVID-19, and all persons aged ≥6 months should stay up to date with recommended COVID-19 vaccination, including receiving ≥1 bivalent vaccine dose.

Before vaccination, providers should provide the EUA Fact Sheet for the vaccine being administered and counsel vaccine recipients about expected systemic and local adverse reactions (reactogenicity). Additional clinical education materials are available,^†††^ including further clinical considerations.^§§§^ These interim recommendations and clinical considerations are based on currently available information regarding bivalent COVID-19 vaccine doses and might change as more evidence becomes available.

## Reporting of Vaccine Adverse Events

Adverse events that occur after receipt of any COVID-19 vaccine should be reported to the Vaccine Adverse Event Reporting System (VAERS, https://vaers.hhs.gov or 1-800-822-7967). Vaccination providers are required by FDA to report vaccine administration errors, serious adverse events, cases of myocarditis, cases of pericarditis, cases of multisystem inflammatory syndrome, hospitalization or death, and cases of COVID-19 that result in hospitalization or death after administration of COVID-19 vaccine under EUA. Reporting is encouraged for any clinically significant adverse event even if it is uncertain whether the vaccine caused the event.

**Summary**

**What is already known about this topic?**

During August–October 2022, CDC recommended a bivalent COVID-19 mRNA vaccine dose for all persons aged ≥5 years who had received a monovalent primary vaccination series.

**What is added by this report?**

During December 2022–April 2023, CDC made recommendations for a single bivalent vaccine dose for most persons aged ≥6 years, bivalent vaccines for children aged 6 months–5 years, and optional additional bivalent booster doses for moderately or severely immunocompromised persons aged ≥6 months and adults aged ≥65 years.

**What are the implications for public health practice?**

Transition to a single bivalent COVID-19 vaccine dose for most persons, with additional doses for persons at increased risk for severe disease, facilitates implementation of simpler, more flexible recommendations. All persons aged ≥6 months should receive ≥1 bivalent vaccine dose.

## Footnotes

* Novavax is authorized as a 2-dose primary series and booster dose in limited situations (e.g., among persons for whom mRNA vaccines are contraindicated or who are unwilling to receive an mRNA vaccine). https://www.cdc.gov/vaccines/covid-19/clinical-considerations/interim-considerations-us.html

†
https://www.cdc.gov/vaccines/hcp/acip-recs/vacc-specific/covid-19.html

§
https://www.cdc.gov/vaccines/acip/recs/grade/downloads/acip-evidence-recs-framework.pdf

¶ https://gis.cdc.gov/grasp/COVIDNet/COVID19_3.html (Accessed May 29, 2023).

** https://covid.cdc.gov/covid-data-tracker/#vaccination-demographics-trends (Accessed June 6, 2023).

†† Among 58 participants aged 6 months–5 years who received 25 *μ*g of the Moderna Omicron BA.1–containing bivalent primary series and had available immunogenicity data, geometric mean ratios of neutralization titers 57 days after the BA.1-containing dose had 25.4 times the Omicron SARS-CoV-2 antibodies compared with titers in those receiving a monovalent dose, meeting superiority criteria. Superiority is considered met when the lower bound of the 97.5% CI of the geometric mean ratio, the ratio of neutralization titers in the intervention versus the control group, is >1.

§§ Safety analysis was conducted among 142 children aged 6 months–5 years with available safety data.

¶¶
https://www.cdc.gov/vaccines/covid-19/clinical-considerations/interim-considerations-us.html#not-immunocompromised

***
https://www.cdc.gov/vaccines/covid-19/clinical-considerations/interim-considerations-us.html#covid-vaccines

†††
https://www.cdc.gov/vaccines/covid-19/index.html

§§§
https://www.cdc.gov/vaccines/covid-19/clinical-considerations/covid-19-vaccines-us.html
